# Controlled Antenatal Thyroid Screening Study III: Effects of Gestational Thyroid Status on Brain Microstructure

**DOI:** 10.1210/clinem/dgaf277

**Published:** 2025-05-09

**Authors:** Carolyn B McNabb, Anna Scholz, Laura Bloomfield, Raghav Bhargava, Charlotte Hales, Colin M Dayan, Sonya Foley, Peter N Taylor, John H Lazarus, Onyebuchi Okosieme, Marian Ludgate, Derek K Jones, D Aled Rees

**Affiliations:** Cardiff University Brain Research Imaging Centre, School of Psychology, Cardiff University, Cardiff CF24 4HQ, UK; Cardiff University Brain Research Imaging Centre, School of Psychology, Cardiff University, Cardiff CF24 4HQ, UK; Centre for Endocrine and Diabetes Sciences, School of Medicine, Cardiff University, Cardiff CF14 4XN, UK; Cardiff University Brain Research Imaging Centre, School of Psychology, Cardiff University, Cardiff CF24 4HQ, UK; Cardiff University Brain Research Imaging Centre, School of Psychology, Cardiff University, Cardiff CF24 4HQ, UK; Centre for Endocrine and Diabetes Sciences, School of Medicine, Cardiff University, Cardiff CF14 4XN, UK; Centre for Endocrine and Diabetes Sciences, School of Medicine, Cardiff University, Cardiff CF14 4XN, UK; Cardiff University Brain Research Imaging Centre, School of Psychology, Cardiff University, Cardiff CF24 4HQ, UK; Centre for Endocrine and Diabetes Sciences, School of Medicine, Cardiff University, Cardiff CF14 4XN, UK; Centre for Endocrine and Diabetes Sciences, School of Medicine, Cardiff University, Cardiff CF14 4XN, UK; Centre for Endocrine and Diabetes Sciences, School of Medicine, Cardiff University, Cardiff CF14 4XN, UK; Centre for Endocrine and Diabetes Sciences, School of Medicine, Cardiff University, Cardiff CF14 4XN, UK; Cardiff University Brain Research Imaging Centre, School of Psychology, Cardiff University, Cardiff CF24 4HQ, UK; Centre for Endocrine and Diabetes Sciences, School of Medicine, Cardiff University, Cardiff CF14 4XN, UK; Neuroscience and Mental Health Innovation Institute, School of Medicine, Cardiff University, Cardiff CF24 4HQ, UK

**Keywords:** thyroid, pregnancy, magnetic resonance imaging, diffusion MRI, quantitative MRI, myelin, attention deficit hyperactivity disorder

## Abstract

**Context:**

Children born to mothers with gestational thyroid dysfunction may have an increased risk of adverse neurodevelopmental outcomes, but the effects of maternal thyroid function on brain microstructure are unknown.

**Objective:**

To establish whether adolescent white matter microstructure is affected by suboptimal gestational thyroid function (SGTF).

**Methods:**

The Controlled Antenatal Thyroid Screening (CATS) study randomized mothers with SGTF to levothyroxine or no supplementation from 12 weeks' gestation. For the current study, CATS children underwent microstructural magnetic resonance imaging (MRI), including diffusion MRI, to explore white matter microstructure and quantitative magnetization transfer (qMT) imaging to investigate myelin. Seventy-five children aged 11-16 years had usable diffusion and/or qMT data: untreated SGTF (n = 19), normal GTF (n = 21), or treated SGTF (optimally treated [n = 18], overtreated [n = 17]). The primary outcome was to examine the effects of SGTF and its treatment on white matter microstructure. Secondary and exploratory outcomes were to investigate the association of (1) maternal thyrotropin and free thyroxine levels with white matter microstructure, and (2) white matter microstructure with attention deficit hyperactivity disorder symptom scores.

**Results:**

Untreated SGTF was associated with higher mean diffusivity (indicating reduced axonal integrity) than normal GTF (*P* = .007) within the inferior longitudinal fasciculus, a major white matter tract connecting the occipital and temporal lobes and involved in several cognitive functions. Secondary and exploratory outcomes did not survive corrections for multiple comparisons.

**Conclusion:**

Untreated SGTF is associated with altered tract-specific microstructural morphology in adolescence, which may be reversible with levothyroxine administration in pregnancy.

Thyroid hormone levels in utero and early childhood are critical for neurodevelopment, as evident by the severe neurological deficits apparent in untreated overt maternal hypothyroidism or congenital hypothyroidism. The impact of less severe, suboptimal gestational thyroid function (SGTF) is less clear, but several studies have reported associations between SGTF and adverse neurobehavioral outcomes, including an increased risk of attention deficit hyperactivity disorder (ADHD) (1-3). These observations mirror findings in rodent models, whereby deficiency of maternal thyroid hormone supply during early gestation can cause disruption of neuronal cell differentiation, proliferation, and myelination (4-7).

While brain morphology may be affected by gestational thyroid availability, human neuroimaging studies are limited. An inverse U-shaped association between maternal thyroid function and gray matter volume was noted in the Generation R cohort, an effect that appeared to be particularly pronounced in early gestation (8, 9). However, in contrast to gray matter, there are few data on white matter development, despite animal and post-mortem data in disorders of reduced thyroid hormone action demonstrating a predominant effect on white matter organization and myelination (4-7, 10). Advances in neuroimaging allow interrogation of white matter in unprecedented detail. Diffusion tensor magnetic resonance imaging (MRI) uses water molecule diffusion to examine white matter microstructure, including integrity and orientation of white matter tracts, while quantitative magnetization transfer imaging (qMT) enables quantification of changes in brain macromolecular density and is used widely to image myelin. Such advances might thus be expected to reveal differences in human white matter structure that have not been apparent in previous studies of altered maternal thyroid hormone exposure.

The CATS (Controlled Antenatal Thyroid Screening) study was a large (n > 22 000) randomized controlled trial of thyroxine supplementation in women with SGTF (11). No differences in IQ were found between the children of untreated and treated mothers with SGTF at age 3 or 9 years (11, 12). However, scores above clinically relevant thresholds for conduct and hyperactivity were significantly more likely in 9-year-old children exposed to high thyroid hormone in utero or overt hypothyroidism (13). In a recent follow-up study of 85 adolescents from the CATS cohort, we found no evidence of an effect of maternal thyroid hormone status on brain morphology at the macrostructural level (14). We therefore hypothesized that altered maternal thyroid hormone availability during neonatal development might affect brain maturation at the microstructural level and sought to investigate this using advanced MRI.

## Materials and Methods

### Participants and Study Design

The current study is a follow-up of the CATS studies, CATS I (11), CATS II (12, 13, 15), and CATS III (14). The original CATS study recruited almost 22 000 women in the UK and Italy between 2002 and 2006 (11), with a primary outcome of child IQ at 3 years of age (11). In CATS II, >400 of the CATS children underwent testing of IQ, neurodevelopment, cardiometabolic, and anthropmetric status at 9 years of age (12, 13, 15). For the current study, we sought to recruit mother–child pairs from each of the 4 study groups: normal gestational thyroid function (normal GTF); untreated SGTF; optimally treated SGTF (free thyroxine [FT4] and thyrotropin [TSH] within reference range at 20 and 30 weeks' gestation); overtreated SGTF, (FT4 > 17.7 pmol/L at either 20 or 30 weeks' gestation as defined by the top 2.5th percentile in the entire CATS UK cohort at consent into the trial) (12). Subjects were recruited between November 2016 and February 2020. Research governance approval was received from Cardiff University (reference SPON 1502-16) and Wales Research Ethics Committee 1 (16/WA/0237). Written and informed consent was obtained from the parents, and assent from the children.

### Eligibility and Investigational Measurements

Eligibility for CATS III has been reported previously (14). Briefly, adolescents were included if they were aged 10-16 years and living sufficiently close to enable return travel to the Cardiff University Brain Research Imaging Centre (CUBRIC) within 1 day. Subjects with contraindications to MRI were excluded from participation. Children completed a validated pubertal self-assessment questionnaire based on the Tanner classification (16). This gives a combined score from a 2-part pictorial questionnaire, with higher scores correlating with more advanced pubertal development.

### MRI Acquisition

Structural (T1 weighted), diffusion, and qMT data were acquired using a 3 Tesla Magnetom Prisma system (Siemens Healthcare, Erlangen, Germany), and a 32-channel receive-only head coil, housed at CUBRIC, Cardiff, UK. Acquisition details for each MRI sequence are provided in the supplemental material (17).

### Preprocessing and Analysis

#### Diffusion data preprocessing and analysis

T1-weighted anatomical data were corrected for field inhomogeneities using the Advanced Normalization Tools (ANTs) (18) and skull stripped using FMRIB Software Library's (FSL version 6.0.5) (19) brain extraction tool. Data were then downsampled to 1.5 mm isotropic voxels.

Diffusion MRI data were corrected for motion and distortions by coregistering the first b0 image to the downsampled T1 image and registering each diffusion MRI volume to the coregistered b0 image using Elastix (20). Gibbs ringing correction was performed using the partial Fourier method (21) and the diffusion tensor for each voxel was estimated with a nonlinear fitting method using the b = 1200 seconds/mm^2^ data (22).

The fiber orientation distribution function for each voxel was calculated using multishell multitissue constrained spherical deconvolution (23). White matter tracts were reconstructed from constrained spherical deconvolution peaks using a combined tract segmentation and orientation mapping approach available in TractSeg (24, 25). We reconstructed 4 major white matter tracts (consisting of 17 individual fiber bundles) previously implicated in the pathogenesis of ADHD (26, 27), including the corpus callosum (7 sections pertaining to the rostrum, genu, rostral body, anterior midbody, posterior midbody, isthmus, and splenium), cingulum (left and right hemispheres), inferior longitudinal fasciculus (ILF; left and right hemispheres), and superior longitudinal fasciculus I-III (SLF-I, SLF-II, SLF-III; left and right hemispheres).

#### QMT preprocessing and analysis

QMT data were correction for motion and distortions using an in house pipeline (details available in supplemental materials) (17). Estimations of qMT parameters providing information on the fractional size of the bound pool were then conducted in QUantitative Imaging Tools (QUIT) (28), implementing the Ramani model (29).

#### Quality control for diffusion MRI and qMT data

To assess the quality of diffusion MRI data, we employed slice-wise outlier detection using SOLID (30), followed by visual assessment of slice drop out in regions of interest. Preprocessed qMT data were visually inspected for hyperintensities, and excessive motion that could not be corrected using traditional motion correction techniques. Any maps deemed unusable were discarded from the analysis.

#### Tractometry

Tractometry was performed in MRtrix3 by computing the median value (fractional anisotropy, mean diffusivity, radial diffusivity, or myelin bound pool fraction) along each streamline for each fiber bundle of interest (17 in total). The mean value for each bundle of streamlines was used in further analysis.

#### Statistical analysis and code availability

Statistical analyses were performed using R statistical software, version 4.4.0 (31). Analysis scripts for this project are available on the Open Science Framework (DOI 10.17605/OSF.IO/FQ4XK). Data were inspected to establish whether they met assumptions for parametric testing; where assumptions were violated, nonparametric tests were used. Influential cases in brain imaging data (identified by Cook's distance, standardized residuals, or leverage values) were excluded from further analysis.

#### Primary analysis: effects of SGTF and treatment on white matter tissue microstructure

The effect of group (normal GTF; untreated SGTF; optimally treated SGTF; and overtreated SGTF) on white matter tissue microstructure (including fractional anisotropy, mean diffusivity, radial diffusivity, and myelin bound pool fraction) was assessed using linear regression. Where a difference between groups was observed, post hoc tests were conducted using pairwise t-tests with correction for multiple comparisons using the Bonferroni method.

#### Secondary analysis: effects of maternal TSH and FT4 on white matter tissue microstructure

The effects of baseline TSH and FT4 levels (measured at entry into CATS I; 12 weeks' gestation) on measures of white matter tissue structure were assessed using partial correlation (accounting for sex, age, and pubertal score). Since TSH and FT4 violated assumptions of normality, Spearman's rank correlation coefficients were used for correlation analyses.

#### Exploratory analysis: relationship between ADHD symptoms and white matter tissue microstructure

Earlier analysis of CATS data suggested that overt maternal hypothyroidism or overtreatment with levothyroxine during gestation increased the risk of ADHD symptoms at age 9 (13). It was therefore of interest to examine whether ADHD symptoms were associated with differences in white matter tissue microstructure in adolescence. The relationship between ADHD symptoms (inattention, overactivity, impulsivity, and overall behavior [total score]) at age 9 and white matter tissue microstructure (fractional anisotropy, mean diffusivity, radial diffusivity, and myelin bound pool fraction) in adolescence was investigated using partial correlation (accounting for sex, age and pubertal score). Since ADHD scores violated assumptions of normality, Spearman's rank correlation coefficients were used for correlation analyses.

#### Exploratory analysis: relationship between IQ and white matter tissue microstructure

Since child IQ was a major focus of the CATS I and CATS II studies (11, 12), we also investigated the relationship between IQ (measured using the Full Scale Intelligence Quotient [FSIQ]) at age 9 (12) and white matter microstructure in adolescence. A partial correlation, (accounting for sex, age, and pubertal status at time of scan) was conducted using a Pearson's correlation (as IQ scores did not violate assumptions of normality).

#### Exploratory analysis: interaction between sex and treatment group across microstructural metrics

Maternal thyroid function has previously been associated with ADHD traits in girls, but not in boys (3). Given the potential association between ADHD symptoms and white matter microstructure in offspring of mothers with maternal thyroid dysfunction, we sought to evaluate whether an interaction between treatment group and sex affects brain tissue microstructure.

Values of *P* < .05 were considered statistically significant for all primary and secondary analyses. Given the nonindependence of fractional anisotropy, mean diffusivity, radial diffusivity, and myelin bound pool fraction, we did not perform corrections for multiple comparisons on these results; this should be considered when interpreting *P* values close to the α threshold of .05.

#### Sample size

Preliminary analysis using 123 Tractometry datasets suggested that a sample size of 80 with a 2-sided α of .05 would provide 80% power to detect a difference of 0.44 SD in myelin water (bound pool) fraction between the normal GTF and SGTF groups. Following quality control, 75 participants' fractional anisotropy data were available for analysis; the smallest sample size per group was 17. A sensitivity analysis conducted using the pwr package (32) in R revealed that our sample size provided 80% power to detect a large effect size (f ≥ 0.41) in an analysis of variance (linear regression) comparing 4 groups. Mean diffusivity, radial diffusivity, and myelin bound pool fraction datasets included 65, 65, and 53 participants, respectively, after removing outliers.

## Results

### Demographic Characteristics

Baseline characteristics of maternal thyroid function during pregnancy as well as characteristics of children at the time of scanning are presented in Table 1. No differences in the proportion of female children, age at time of scan or Tanner pubertal score were observed between groups. By design, levels of TSH were higher and FT4 were lower in the SGTF groups than in normal GTF group at study entry (12 weeks' gestation). At 20 and 30 weeks' gestation, FT4 levels were higher in the overtreated SGTF group than the optimally treated SGTF group.

**Table 1.** Baseline characteristics of the CATS III study cohort

	Normal GTF (n = 21)	Optimally treated SGTF (n = 18)	Overtreated SGTF (n = 17)	Untreated SGTF (n = 19)	Test statistic; *P* value
Female children (%)	57	50	47	42	H = .935; *P* = .817
Age of children (years)	13.7 (2.5)	14.9 (2.7)	13.0 (1.8)	13.7 (1.7)	H = 7.69; *P* = .053
Pubertal score	6.0 (2.0)	8.0 (1.0)*^d^*	6.0 (1.5)*^d^*	7.0 (3.0)	H = 3.85; *P* = .278
TSH at 12 weeks (study entry; mlU/L)	1.0 (0.8)*^c^*	3.2 (2.9)*^b^*	4.5 (1.1)*^b^*	3.6 (4.2)*^a^*	H = 28.9; *P* < .001
TSH at 20 weeks (mlU/L)	—	0.3 (0.9)	0.1 (0.4)	—	H = 2.31; *P* = .128
TSH at 30 weeks (mlU/L)	—	0.2 (0.4)	0.2 (0.3)	—	H = .829; *P* = .363
FT4 at 12 weeks (study entry; pmol/L)	14.0 (1.0)*^c^*	10.6 (3.5)*^a^*	12.5 (3.4)	10.8 (1.9)*^b^*	H = 18.6; *P* < .001
FT4 at 20 weeks (pmol/L), mean (SD)	—	15.7 (1.2)	18.5 (2.2)	—	F = 22.7; *P* < .001
FT4 at 30 weeks (pmol/L)	—	14.6 (1.7)	16.9 (2.1)	—	H = 11.2; *P* < .001

Values are reported as median (interquartile range), unless otherwise stated.

Group comparisons were conducted using the Kruskal–Wallis H test (Wilcoxon rank sum test for post hoc comparisons) and analysis of variance (F test).

Abbreviations: GTF, gestational thyroid function; SGTF, suboptimal gestational thyroid function; TSH, thyroid stimulating hormone; FT4, free thyroxine.

^*a*^ *P* < .01 vs normal GTF.

^*b*^ *P* < .001 vs normal GTF (post hoc pairwise Wilcoxon test, Bonferroni corrected).

^*c*^Missing thyroid function tests, n = 1.

^*d*^Missing total Tanner scores, n = 1 optimally treated SGTF and n = 2 overtreated SGTF.

### Primary Analysis: Effects of SGTF and Treatment on White Matter Tissue Microstructure

**Figure 1.**
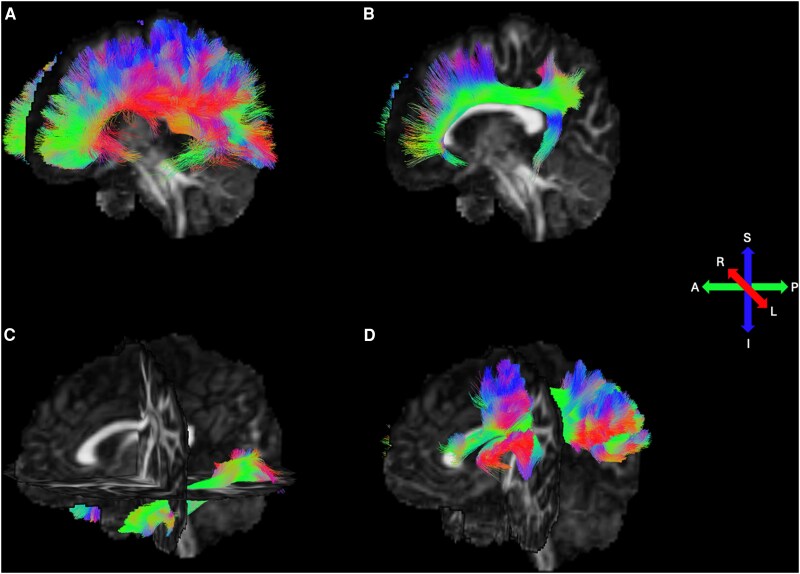
Major white matter tracts of interest for CATSIII. (A) Corpus callosum (consisting of bundles I-VII); (B) cingulum; (C) inferior longitudinal fasciculus; (D) superior longitudinal fasciculus (consisting of bundles I-III). Colors represent the principal fiber direction: red, left to right; green, anterior to posterior; blue, inferior to superior. Tracts visualized using Fibernavigator (https://scilus.github.io/fibernavigator/).

Figure 1 depicts the 4 major white matter tracts (corpus callosum, cingulum, ILF, and SLF) of interest, reconstructed using Tractseg.

**Figure 2.**
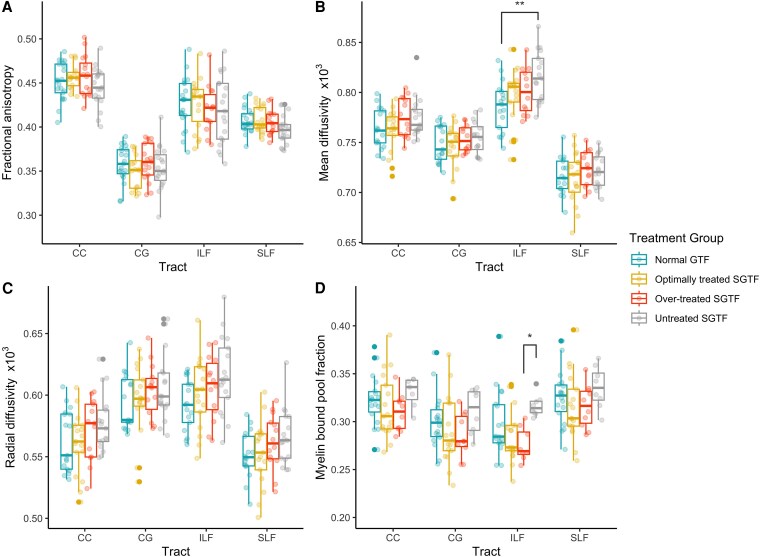
Effect of maternal thyroid function on white matter tissue microstructure in adolescent children. CC, corpus callosum; CG, cingulum; ILF, inferior longitudinal fasciculus; SLF, superior longitudinal fasciculus. *P* values are corrected for multiple comparisons using the Bonferroni method. *P < .05; **P ≤ .01 in post hoc pairwise t test.

The effects of maternal thyroid function on white matter tissue microstructure in the 4 tracts of interest are shown in Fig. 2. Mean diffusivity in the ILF was highest in children of mothers with untreated SGTF, with a statistically significant difference observed between untreated SGTF and normal GTF (*F*(3,59) = 4.00, *P* = .012; post hoc Bonferroni corrected pairwise t test *P* = .007). Within the same tract, myelin bound pool fraction was also highest in children of mothers with untreated SGTF, with a statistically significant difference observed between untreated SGTF and overtreated SGTF (*F*(3,47) = 3.787, *P* = .016; post hoc Bonferroni corrected pairwise t test *P* = .027). No other statistically significant differences were observed.

**Figure 3.**
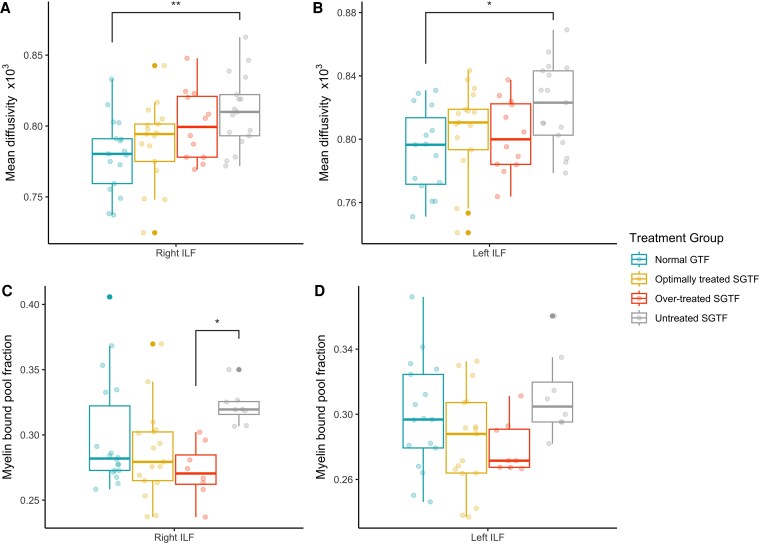
Effect of maternal thyroid function on white matter tissue microstructure in the ILF in adolescent children. ILF, inferior longitudinal fasciculus. *P* values are corrected for multiple comparisons using the Bonferroni method. *P < .05; **P ≤ .01 in post hoc pairwise t test.

A post hoc analysis was conducted to determine whether the effect of maternal thyroid function on the microstructural tissue properties of the ILF was present in both hemispheres or lateralized to the left or right hemisphere of the brain. Results are presented in Fig. 3. The effect of maternal thyroid function on mean diffusivity was present in both left and right ILF, with statistically significant differences observed between the untreated SGTF and normal GFT groups in both cases (right ILF: *F*(3,59) = 4.32, *P* = .008; post hoc Bonferroni corrected pairwise t test *P* = .008; left ILF: *F*(3,57) = 4.32, *P* = .028; post hoc Bonferroni corrected pairwise t test *P* = .021). The effect of maternal thyroid function on myelin bound pool fraction was confined to the right ILF (*F*(3,47) = 3.36, *P* = .026; post hoc Bonferroni corrected pairwise t test *P* = .030); however, the pattern across groups was similar in both hemispheres.

### Secondary Analysis: Effects of Maternal Thyroid Stimulating Hormone and Free Thyroxine on White Matter Tissue Microstructure

Baseline levels of TSH (measured at 12 weeks' gestation) were significantly positively correlated with mean diffusivity in the ILF (ρ=0.310, uncorrected *P* = .015). Given the large number of correlational tests performed, this effect would not survive corrections for multiple comparisons and should be interpreted with care. No other statistically significant effects of maternal TSH or FT4 on adolescent white matter tissue microstructure were observed (Table S1 (17)).

### Exploratory Analysis: Relationship Between ADHD Symptoms and White Matter Tissue Microstructure

High impulsivity was negatively correlated with fractional anisotropy in the corpus callosum (ρ=−0.290, uncorrected *P* = .015) and overactivity was positively correlated with mean diffusivity in the ILF (ρ=0.287, uncorrected *P* = .025). Again, given the large number of correlational tests performed, these effects would not survive corrections for multiple comparisons and should be interpreted with care. No other statistically significant relationships between ADHD scores and measures of white matter tissue microstructure were observed (Table S2 (17)).

### Exploratory Analysis: Relationship Between IQ and White Matter Tissue Microstructure

No statistically significant relationships between FSIQ scores at age 9 and measures of white matter tissue microstructure in adolescence were observed (Table S3 (17)).

### Exploratory Analysis: Interaction Between Sex and Treatment Group Across Microstructural Metrics

Linear regression analyses exploring the relationship between treatment group, sex, their interaction, and each microstructural metric within each tract of interest revealed effects within the ILF for mean diffusivity, radial diffusivity, and myelin bound pool fraction, and within the SLF for mean diffusivity (Table S4 (17)). Correction for multiple comparisons using the false discovery rate rendered all effects not statistically significant (Table S4 (17)). For exploratory purposes, regression analyses exhibiting uncorrected *P* < .05 were further analyzed using a series of post hoc t tests, also corrected using the false discovery rate (Table S5 (17)). No test survived correction for multiple comparisons, though a boxplot showing differences between male and female microstructural metrics across treatment groups and tracts using uncorrected *P* values is provided for completeness (Fig. S1 (17)).

## Discussion

In this follow-up study of the CATS randomized trial, we found evidence of altered white matter microstructure among adolescents born to mothers with untreated SGTF. These alterations were confined to the ILF, which was unaffected in adolescents whose mothers had been treated antenatally with levothyroxine. Our observations suggest that untreated SGTF is associated with altered tract-specific microstructural morphology in adolescence, which may be reversible with levothyroxine administration in pregnancy.

Maternal thyroid hormone exposure plays a critical role in optimizing human neurodevelopment, as evidenced by the profound impact on offspring cognition and motor function of untreated overt maternal hypothyroidism (33). Maternal thyroid hormone is the only source of fetal thyroid exposure until midgestation, and continues to play an important role throughout pregnancy while the fetal thyroid gland reaches full maturity. In rodents, experimental hypothyroidism in early gestation has been shown to disrupt neuronal proliferation, differentiation and migration, neurite outgrowth, synaptogenesis, and myelination (4, 5). These deficits are evident even if the reduction in maternal thyroid hormone exposure is only transient or moderate, and may only be reversible with levothyroxine administration if treatment is commenced quickly after the induction of hypothyroidism (6, 7). In humans, thyroid hormone receptor expression in the fetal brain increases 10-fold between 10 and 16 weeks' gestation (34), coincident with a time of rapid neuroblast proliferation and fetal axonal development (35). Observational data of adverse cognitive outcomes in children born to mothers with untreated SGTF (36) suggest that fetal compensatory mechanisms may be insufficient to overcome reduced maternal thyroid hormone bioavailability.

Our findings extend our previous work, in which we found no evidence of altered brain morphology among adolescents born to mothers with normal or SGTF, nor an effect of treatment with levothyroxine (14). Our previous analyses focused on brain macrostructure, using global volumetric analysis of gray and white matter. Our current observations thus imply that white matter microstructural indices are a more sensitive measure of thyroid hormone action in the developing human brain, and that perturbations in maternal thyroid hormone status may affect white matter integrity more than volume. Most previous studies examining the association between maternal thyroid status and childhood brain morphology used a volumetric MRI approach (8, 9, 37). In analyses of children from the Generation R cohort, an inverse U-shaped association between maternal thyroid function and gray matter volume was noted (8), a relationship which was most pronounced in early gestation (9).

A strength of our study is that we combined quantitative magnetization transfer imaging with “traditional” measures of white matter microstructure (mean diffusivity, radial diffusivity, and fractional anisotropy) in order to interrogate any effect on myelin as well as axonal structure. Mean diffusivity represents the overall directionally averaged magnitude of diffusion. An increased mean diffusivity, as observed here, is thus expected to reflect reduced white matter integrity from either axonal or myelin degradation. Bound pool fraction mapping quantifies exchanging protons bound to macromolecules (such as myelin) and is considered a more direct measure of myelin content. Since the myelin bound pool fraction was not different between the untreated SGTF and normal GTF groups, this therefore implies that the observed increase in mean diffusivity is not attributable to myelin degradation but rather to reduced axonal integrity. Furthermore, the preservation of myelin suggests that a compensatory response may be in operation, such as an increase in fetal thyroid hormone production in later pregnancy. Such an effect would be consistent with our understanding of myelination as a later step in maturation of the human fetal brain (38, 39), and with rodent data which show that transient hypothyroidism stimulates the production of oligodendrocytes (the myelinating cells within the central nervous system), leading to subsequent remyelination (40). Remyelination may be consolidated by triiodothyronine, which is important in oligodendrocyte differentiation, maturation, and myelination (41-43). In a secondary analysis, we found that maternal TSH at baseline (12 weeks' gestation) was positively correlated with mean diffusivity in the ILF. Although this finding should be interpreted with caution since it would unlikely survive correction for multiple comparisons, this observation is nevertheless in keeping with our primary finding of an effect of SGTF on white matter integrity in this tract.

The effects of SGTF on white matter microstructure appeared to be tract specific in our study, with no differences evident between groups in any microstructural indices in the SLF, cingulum, or corpus callosum. By design, we focused our attention on white matter tracts that have been implicated in the pathogenesis of ADHD, this based on our previous findings of increased hyperactivity scores at age 9 in CATS children exposed to overtreatment with levothyroxine or overt hypothyroidism (13). Although it is conceivable that we may have missed an effect in other white matter tracts, our observation of an increased mean diffusivity in the ILF in adolescents born to SGTF mothers implies that this tract may be especially sensitive to altered in utero thyroid hormone bioavailability. The ILF is recognized as a multifunctional tract involved in visual perception (44) and the ventral semantic system for language (45). A previous diffusion tensor imaging study of the human fetal brain showed that the ILF develops predominantly during the second trimester (35), a time period that coincides with ongoing fetal dependence on adequate maternal thyroid hormone exposure. Altered white matter microstructure in the ILF has previously been demonstrated in children and adults with ADHD, and in hypothyroid adults compared with age-matched controls (46). Weaker functional connectivity of the lingual gyrus to the insula, both of which have connections with the ILF, has also been demonstrated in patients with resistance to thyroid hormone beta, who exhibit behavioral symptoms akin to ADHD (47). Furthermore, the ILF was 1 of several white matter tracts showing altered microstructure in patients with permanent congenital hypothyroidism and lower IQ and perceptual reasoning scores (48). In our exploratory analysis, which should be interpreted with caution, we found a significant correlation of overactivity score at age 9 with mean diffusivity in the ILF. Bjornhölm et al examined the association of maternal thyroid function during early pregnancy with offspring white matter integrity in early adulthood in 292 mother–child pairs in the 1986 Northern Finland Birth Cohort (NFBC) (49). They found that higher maternal FT4 was associated with greater white matter integrity in males, as determined by global analysis of fractional anisotropy in 14 white matter tracts. This observation was driven by multiple white matter tracts, of which 4 (corticospinal tract, anterior and posterior thalamic radiation, forceps minor) survived correction for multiple comparisons (49). In agreement with our findings, they did not find an association between maternal thyroid function and myelin, as measured by magnetization transfer ratio, again implying an influence of maternal thyroid status on axonal rather than myelin development. However, in contrast to our study, they found no association between maternal thyroid function and ILF microstructure. Potential explanations for this difference may lie in the analysis approach (global followed by tract-wise analysis [NFBC] vs tract-specific analysis [CATS]), population sampled (unselected [NFBC] vs predominantly SGTF [CATS]), and age of the offspring (28 years [NFBC] vs 13 years [CATS]), but not in the timing of blood sampling, which was similar in both studies (11 weeks' [NFBC] vs 12 weeks' [CATS] gestation). Bjornhölm et al's observations of an effect in male but not female offspring suggests that maternal antenatal thyroid function may affect white matter development in a sex-specific manner. We therefore undertook an exploratory analysis to examine if any interaction was found between treatment group and sex, finding no significant influence of sex on the effect of treatment group on any microstructural measure. Recognizing that previous functional anatomy studies have shown that there may be different roles attributable to the right and left ILF (50), we also undertook a post hoc analysis to explore each tract individually, finding no evidence that the effect lateralized to either hemisphere.

In contrast to untreated SGTF, all white matter microstructural indices among adolescents born to mothers who were randomized to treatment with levothyroxine (whether optimally replaced or over-replaced) were not different from the normal GTF group. This observation suggests that there may still be a benefit of levothyroxine in the treatment of SGTF even though 2 large randomized, controlled trials of levothyroxine supplementation have failed to demonstrate an improvement in child IQ (11, 51). Although a number of explanations have been proposed to explain these findings (36), our study suggests that diffusion tensor imaging may offer a more sensitive and objective measure of treatment response. Nevertheless, more studies are needed before a recommendation can be made for routine levothyroxine supplementation in SGTF, and caution needs to be exercised with respect to dose monitoring in view of our previous observations of an increased risk of adverse neurobehavioral outcomes in mothers who had been over-replaced (13).

Our study has a number of strengths, including the application of advanced MRI to interrogate axonal and myelin metrics, and inclusion of children whose mothers had received levothyroxine supplementation in pregnancy within a randomized clinical trial design. We also adjusted for the potential confounding influences of puberty, age, and sex on white matter maturation. However, our study also has several limitations, including the lack of repeat neurobehavioral assessment, which may have offered insights into the persistence or otherwise of the adverse neurobehavioral traits that we had observed at age 9, in addition to contemporaneous comparison of questionnaire scores with microstructural indices. Furthermore, we did not assess adolescent thyroid function, did not have access to birth outcomes with the potential to also affect brain microstructure (eg, preterm delivery, neonatal hypoxia, birth weight), and participants were scanned only once, hence we are unable to offer insight into any potential effect of maternal thyroid status on the trajectory of axonal and myelin development.

In conclusion, we found evidence of altered tract-specific white matter microstructure in adolescents born to mothers with SGTF, in this follow-up of participants from the CATS study. Microstructure was unaffected in adolescents whose mothers had been supplemented with levothyroxine during pregnancy. While caution is needed to avoid overinterpretation of these findings so as not to cause unnecessary patient anxiety, our observations underscore the value of diffusion MRI as a sensitive measure of thyroid hormone action in the human brain, and suggest that levothyroxine supplementation may restore normal brain morphology despite the absence of evidence at present for a benefit on cognition. Further studies are needed to replicate our findings, to establish the mechanism(s) of this effect, and to determine whether differences in offspring brain microstructure in SGTF are accompanied by clinical sequelae such as suboptimal educational attainment or an increased risk of neurodevelopmental disorders.

## Data Availability

The data that support the findings of this study are available from the corresponding author upon reasonable request. Analysis scripts for this project are available on the Open Science Framework (OSF; https://osf.io/fq4xk/).

## Abbreviations

ADHD: attention deficit hyperactivity disorder
CATS: Controlled Antenatal Thyroid Screening
FT4: free thyroxine
GFT: gestational thyroid function
ILF: inferior longitudinal fasciculus
MRI: magnetic resonance imaging
qMT: quantitative magnetization transfer
SGTF: suboptimal gestational thyroid function
SLF: superior longitudinal fasciculus
TSH: thyrotropin
